# The novel β-TrCP protein isoform hidden in circular RNA confers trastuzumab resistance in HER2-positive breast cancer

**DOI:** 10.1016/j.redox.2023.102896

**Published:** 2023-09-28

**Authors:** Shengting Wang, Yufang Wang, Qian Li, Xiaoming Li, Xinghua Feng, Kaixuan Zeng

**Affiliations:** a Clinical Medical Center, Xi'an Peihua University, Xi'an, 710125, Shaanxi, China; b Precision Medical Research Institute, The Second Affiliated Hospital of Xi'an Jiaotong University, Xi'an, 710000, Shaanxi, China

**Keywords:** Circular RNA, Protein coding, Trastuzumab resistance, NRF2, Protein degradation

## Abstract

Trastuzumab notably improves the outcome of human epidermal growth factor receptor 2 (HER2)-positive breast cancer patients, however, resistance to trastuzumab remains a major hurdle to clinical treatment. In the present study, we identify a circular RNA intimately linked to trastuzumab resistance. *circ-β-TrCP*, derived from the back-splicing of *β-TrCP* exon 7 and 13, confers trastuzumab resistance by regulating NRF2-mediated antioxidant pathway in a KEAP1-independent manner. Concretely, *circ-β-TrCP* encodes a novel truncated 343-amino acid peptide located in the nucleus, referred as β-TrCP-343aa, which competitively binds to NRF2, blocks SCF^β-TrCP^-mediated NRF2 proteasomal degradation, and this protective effect of β-TrCP-343aa on NRF2 protein requires GSK3 activity. Subsequently, the elevated NRF2 transcriptionally upregulates a cohort of antioxidant genes, giving rise to trastuzumab resistance. Moreover, the translation ability of *circ-β-TrCP* is inhibited by eIF3j under both basal and oxidative stress conditions, and eIF3j is transcriptionally repressed by NRF2, thus forming a positive feedback circuit between β-TrCP-343aa and NRF2, expediting trastuzumab resistance. Collectively, our data demonstrate that *circ-β-TrCP*-encoded β-TrCP protein isoform drives HER2-targeted therapy resistance in a NRF2-dependent manner, which provides potential therapeutic targets for overcoming trastuzumab resistance.

## Highlights

• Circ-β-TrCP is upregulated in trastuzumab-resistant HER2-positive breast cancer.
• Circ-β-TrCP promotes trastuzumab resistance.
• Circ-β-TrCP encodes β-TrCP-343aa, which is repressed by eIF3j.
• β-TrCP-343aa blocks SCF^β-TRCP^-mediated degradation of NRF2.
• NRF2 increases the translation efficiency of circ-β-TrCP via transcriptional inhibition of eIF3j.

## Graphical abstract

**Figure:**
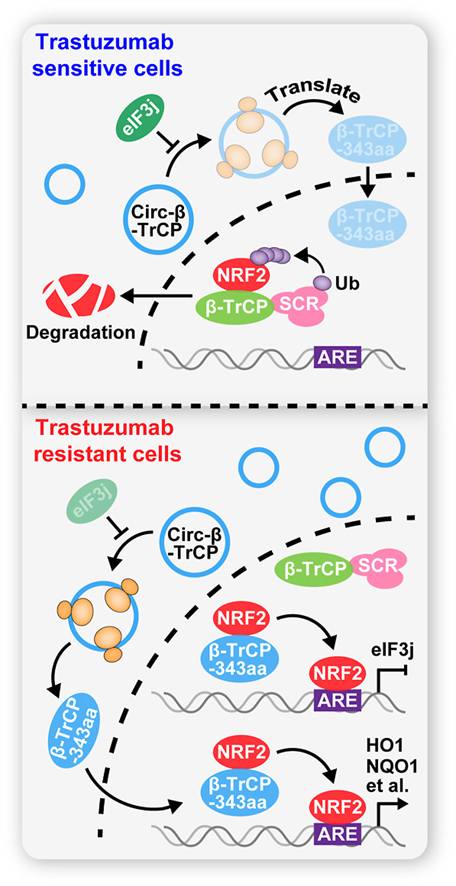

## 1 Introduction

Breast cancer is the most common malignancy among women, the number of new cases and deaths of breast cancer ranks first in the world, accounting for 25% of the total female cancer cases and 17% of the cancer deaths [1]. As a special subtype, human epidermal growth factor receptor 2 (HER2)-positive breast cancer accounts for 15–20% of all breast cancer, which is characterized by aggressive behaviors and poor prognosis [2]. Trastuzumab, a humanized monoclonal antibody, exhibits considerable anti-tumor efficacy in both preclinical and clinical trials [3]. It is initially approved by the FDA for treatment of HER2-positive breast cancer in 1998, and patients receiving trastuzumab treatment have prolonged overall survival time of nearly five months [4]. However, a high proportion of patients eventually develop resistant to trastuzumab [5], which enormously limits its clinical application. Therefore, an improved understanding of the potential mechanism of HER2-targeted therapy resistance will allow identification of new therapeutic strategies to overcome resistance.

The adaptation of tumor cells to oxidative stress is responsible for the insensitivity to anti-tumor drugs [6]. NRF2, a well-known basic leucine zipper transcription factor, acts as a master regulator of redox homeostasis via governing the expression of hundreds of genes involved in cell defense against oxidative stress [7]. It is clear that NRF2 protein turnover is tightly controlled by the ubiquitin-proteasome system involving KEAP1-Cul3-Rbx1 complex in the cytoplasm and SKP1-Cul1-Rbx1 (SCF^β-TRCP^) complex in the nucleus [8]. β-TRCP directly binds to NRF2 through its WD domain, while recruits SKP1-Cul1-Rbx1 complex through its F-box domain, resulting in NRF2 ubiquitination and degradation, and the binding of β-TRCP to NRF2 requires the phosphodegron created by GSK-3 [9]. Extensive evidence suggests that NRF2 is frequently overexpressed in human cancer and contributes to drug resistance by preventing oxidative stress [10]. Recent studies showed that HER2-amplified patients with high NRF2 expression had worse progression-free survival following trastuzumab treatment [11]. And brusatol, a novel Nrf2 inhibitor, was capable to synergistically potentiate the anti-tumor activity of trastuzumab in HER2-positive cancers [12], indicating that NRF2 is critical for trastuzumab resistance.

Circular RNA is a type of single strand endogenous closed loop RNA that lacks 3'-end poly (A) tail and 5'-end cap structure [13]. It is mainly produced by precursor mRNA (pre-mRNA) through back splicing processing, and is highly abundant in eukaryotes, with some circRNAs being expressed at more than 10-fold higher levels than their parental linear mRNAs [14]. Due to the limitations of detection technology, circRNA is initially considered as transcriptional “noise” and has long been overlooked by researchers. Nowadays, circRNA is widely recognized as a pivotal regulator in pathological and physiological changes [15,16]. The mechanism of action of circRNA is complex, including acting as microRNA sponge, regulating gene expression and selective splicing, and binding to various proteins [17]. Of note, genome-wide translatomics suggests that circRNA is translatable in human cells [18], and a portion of circRNAs have been proven to be directly translated into the functional proteins, participating in cancer occurrence, development and progression [19,20]. For instance, the novel 370-amino acid β-catenin isoform encoded by *circ-β-catenin* promoted hepatocellular carcinoma growth through stabilizing full-length β-catenin protein [21]. The micropeptide E-Cad-254aa translated by *circ-E-Cad* maintained glioma stem cell tumorigenicity through activation of EGFR-STAT3 signaling [22]. In addition, *circ-AXIN1*-encoded AXIN1-295aa competitively interacted with APC, resulting in activation of Wnt signaling pathway in gastric cancer cells [23]. These studies indicate that the previously unrecognized endogenous micropeptides translated by circRNAs harbor important biological functions, but whether they are involved in trastuzumab resistance remains unknown.

Herein, we found a circular RNA produced by *β-TrCP* promoting trastuzumab resistance via encoding a novel β-TrCP isoform, β-TrCP-343aa. Further investigations revealed that β-TrCP-343aa functioned as a stabilizer of NRF2 protein through blunting SCF^β-TRCP^-mediated NRF2 ubiquitination degradation. Moreover, we found that the translation efficiency of *circ-β-TrCP* was altered in response to oxidative stress, a process controlled by the NRF2/eIF3j axis.

## 2 Materials and methods

### 2.1 Tissue samples

The fresh surgically excised specimens were obtained from The Affiliated Shaanxi Fourth People Hospital of Peihua University. HER2-positive breast cancer was determined by immunohistochemistry (IHC) staining, and fluorescence in situ hybridization (FISH) was required when IHC result was intermediate staining intensity. We collected a total of 80 tissues, of which 30 were sensitive to trastuzumab and 50 were insensitive. The detailed clinicopathological information is described in Table S1. Resistance to trastuzumab was defined as progression at the first radiological reassessment within 8–12 weeks or 3 months after first-line trastuzumab with or without chemotherapy, or as a new diagnosed recurrence within adjuvant trastuzumab therapy for 12 months [24]. In addition, 15 trastuzumab-sensitive and 18 trastuzumab-resistant plasma specimens were also obtained from above patients. All patients were routinely followed up and provided written informed consent. And this study was conducted in line with the Declaration of Helsinki, and approved by the Ethics Committee of The Affiliated Shaanxi Fourth People Hospital of Peihua University.

### 2.2 Cell lines

HEK-293T and HER2-positive breast cancer cell lines BT474 and SKBR3 were obtained from Cell Bank of Chinese Academy of Sciences. Cells were maintained in DMEM medium supplemented with 10% fetal bovine serum (Gibco, USA) and tested for mycoplasma contamination every 3 months. Establishment of cell lines resistant to trastuzumab was conducted by gradually increasing trastuzumab concentrations as described in our previous study [25]. Trastuzumab-resistant BT474 (BT474-TR) and SKBR3 (SKBR3-TR) cells were cultured in DMEM medium supplemented with 15 μg/mL trastuzumab. For the functional assays, trastuzumab was removed for at least one week to avoid acute effects.

### 2.3 Quantitative reverse-transcription PCR (qRT-PCR)

Total RNA was isolated by 1 mL Trizol reagent (Invitrogen, USA), followed by measurement of RNA concentration using NanoDrop™ spectrophotometers (Thermo Scientific, USA). 1 μg RNA was reverse transcribed to cDNA using PrimeScript^RT^ Master Mix (Takara Bio, Japan) as per manufacturer's instructions. Then, cDNA was diluted 5-fold, nucleic acid amplification and quantification were conducted using SYBR Premix^Ex^ Taq Kit (Takara Bio) and ABI7900 fast real-time PCR system (Applied Biosystems, USA). Gene expression was determined by 2^−ΔΔCt^ formula, and ACTB gene was used as the reference control. The primer sequences are listed in Table S2.

### 2.4 Northern blot

The assay was conducted by using NorthernMax™ Kit (Thermo Scientific) according to manufacturer's instructions with minor modifications. In brief, 10 μg total RNA was treated with 5 U/μg RNase R (Epicentre Technologies, USA) at 37 °C for 20 min, separated on 1.5% denaturing agarose gel and transferred to hybond-N^+^ membrane. Then, the membrane was hybridized with biotin-labeled probe targeting *circ-β-TrCP* junction site at 58 °C overnight with gentle rotation. The next day, the membrane was rigorously washed and incubated with HRP-linked streptavidin for 30 min at room temperature. The signal on the membrane was detected using Chemiluminescent Nucleic Acid Detection Module Kit (Thermo Scientific). The oligonucleotide probe sequences used in northern blot assay are listed in Table S2.

### 2.5 Detection of *circ-β-TrCP* subcellular localization

Isolation of cytoplasmic and nuclear RNA was carried out using Ambion® PARIS™ Kit (Thermo Scientific) as per manufacturer's instructions. *ACTB* and *U6* were used as cytoplasmic and nuclear control fragments, respectively. For FISH assay, the Cy3-labeled probe against *circ-β-TrCP* junction site was designed and synthesized by GenePharma (Shanghai, China), followed by incubation with cell lysates. The assay was conducted using RNA FISH Kit (GenePharma) as per manufacturer's instructions. Cell nucleus was stained by DAPI dye, and the fluorescent signals were observed under a fluorescent microscope. The oligonucleotide probe sequences used in FISH assay are listed in Table S2.

### 2.6 Vectors, siRNAs and transfection

The CRISPR/Cas13d system [26] was used to establish *circ-β-TrCP* knockdown cells *in vivo*. Three sgRNAs targeting *circ-β-TrCP* junction site were designed and inserted into pLKO.1 vector containing direct repeats of RfxCas13d, followed by lentivirus packaging using psPAX2 and pMD2.G vectors. The stable cell lines were screened by 1.5 μg/mL puromycin. To overexpress *circ-β-TrCP*, the full-length of *circ-β-TrCP* was synthesized and inserted into pLV-circ-Puro plasmid with two reverse complementary sequences, followed by lentivirus packaging and infection into cells. To overexpress genes, the full-length coding sequences of human *eIF3j*, *β-TrCP*, *NRF2*, *β-TrCP-343aa* were cloned into pcDNA 3.1, pFlag-CMV-5a and pCMV-HA plasmids as appropriate. The various mutated vectors were prepared using Q5® Site-Directed Mutagenesis Kit (New England Biolabs, UK) as per manufacturer's protocols. To construct NRF2 knockout cells, the validated sgRNA targeting *NRF2* was inserted into lenti-CRISPR v2 plasmid, followed by lentivirus packaging using psPAX2 and pMD2.G vectors and infection into cells. The stable cell lines were screened by 1.5 μg/mL puromycin. The knockout efficiency was verified by Sanger sequencing and Western blot assay. To generate endogenous *eIF3j* antioxidant response element (ARE)-mutated cells, the designed sgRNA with the lowest rate of off-target score was synthesized and ligated into pSpCas9(BB)-2A-Puro plasmid, followed by electroporation along with single-stranded oligodeoxynucleotide (ssODN) into SKBR3 cells using Lonza Amaxa 4D-Nucleofector as per the manufacturer's instructions. The limited dilution method was used to obtain individual colony in 96-well plates. The mutated efficiency was verified by Sanger sequencing. All siRNAs were designed and synthesized by Sigma-Aldrich, followed by transfection into cells using RiboJuice siRNA Transfection Reagent (Sigma-Aldrich, USA). And plasmid transfection was conducted using Lipofectamine 3000 (Invitrogen). The sgRNA, siRNA and ssODN sequences used in this study are listed in Table S2.

### 2.7 CCK-8, colony formation and flow cytometry

Cell viability was determined by CCK-8 assay. Equal amounts of cells were seeded into 96-well plates, and when grown to 70–80% confluence, 10 μL CCK-8 reagent was added into the medium. After incubation for 2 h at 37 °C, the absorbance at 450 nm in each well was detected by an automatic microplate spectrophotometer. For colony formation assay, 3000 cells were seeded into 6-well plates, and cultured for 2 weeks. Then, cells were fixed by methanol and stained by crystalline violet. ROS content was detected by H2DCFDA probe (MedChemExpress, USA) and CellROX Deep Red reagent (Invitrogen). In brief, equal amounts of cells were seeded into 6-well plates, and when grown to 70–80% confluence, cells were washed by PBS and incubated with 20 μM H2DCFDA probe at 37 °C for 1 h. Then, cells were collected and resuspended in 300 μL PBS, followed by analysis using Accuri™ C6 Plus flow cytometer (BD Biosciences, USA). For CellROX staining, cells were plated into 96-well plates, followed by incubation with 5 μM CellROX Deep Red reagent at 37 °C for 30 min. After washing twice with PBS, the fluorescence signals were detected by using the CLARIOstar® Plus microplate reader (BMG LABTECH, Germany).

### 2.8 Orthotopic transplantation tumor model

A total of 1 × 10^7^ *circ-β-TrCP*-silenced BT474-TR cells or *circ-β-TrCP*-overexpressed BT474 cells mixed with 1:1 matrigel (Corning, USA) were orthotopically injected into the abdominal mammary fat pad of NOD/SCID mice grown under specific-pathogen-free condition. When the tumor grew to 0.05–0.1 cm^3^ in size, mice were treated with vehicle or trastuzumab (20 mg/kg, intraperitoneal administration) once a week. Tumor volume was recorded using a vernier caliper and calculated using the formula: 1/2 × length × width^2^, no mice died during the experiment. At the end of the fourth week, all mice were sacrificed and tumor tissues were collected, weighed and photographed. The animal study was approved by the Animal Care and Use Committee of Peihua university.

### 2.9 Western blot, co-immunoprecipitation (Co-IP) and IHC staining

The protocols were as previously described [27]. Briefly, cells were trypsinized and washed twice with ice-cold PBS, followed by treatment with lysis buffer containing 0.5 M Tris-HCl, 1.5 M NaCl, 10% NP-40, 10 mM EDTA, 2.5% deoxycholic acid, and protease inhibitor cocktail (Roche, USA). Total protein was quantified using Pierce Rapid Gold BCA Protein Quantitative Kit (Thermo Fisher Scientific) and loaded to SDS-PAGE gel and transferred to PVDF membrane. After blocking with 5% nonfat milk, the membrane was incubated with the corresponding primary and secondary antibodies, and developed using SuperSignal West Atto reagent (Thermo Fisher Scientific). For Co-IP assay, the corresponding affinity gels or antibodies and protein A/G magnetic beads (Thermo Fisher Scientific) were added into cell lysates and incubated for 5 h at 4 °C with gentle rotation. The beads were extensively washed and resuspended in Laemmli buffer, followed by Western blot assay. For *in vivo* ubiquitylation assay, cells were treated with 20 μM MG132 (MedChemExpress) before collection of cell lysates, followed by Co-IP assay. IHC staining was carried out using SP Rabbit & Mouse HRP Kit (DAB) (Cowin, China) according to manufacturer's instructions. Assessment of IHC staining was conducted by using H-score method as previously described [28]. The antibodies used in this study are listed in Table S3.

### 2.10 RNA pull-down and immunoprecipitation (RIP)

For *in vitro* pull-down assay, the linear *circ-β-TrCP* was *in vitro* transcribed and labeled with biotin, followed by circularization using T4 RNA ligase I (New England Biolabs). Then, the purified eIF3j protein (#Ag0678, Proteintech, USA) was incubated with *circ-β-TrCP* probe, followed by Western blot assay. For *in vivo* pull-down assay, the biotin-conjugated probe against the junction site of circ-β-TrCP was synthesized and incubated with cell lysates at 4 °C for 5 h. Then, the streptavidin magnetic beads (Invitrogen) were added and incubated for another 1 h. The beads were extensively washed and resuspended in Laemmli buffer, followed by Western blot assay. The oligonucleotide probe sequences are listed in Table S2. RIP assay was conducted using Magna RIP Kit (Millipore, USA) according to manufacturer's instructions, followed by qRT-PCR analysis of circ-β-TrCP enrichment.

### 2.11 Luciferase reporter assay

BT474-TR and SKBR3-TR cells were transfected with 500 ng ARE-Firefly luciferase or pGL3-basic-*eIF3j*-promoter plasmid along with 100 ng pRL-CMV Renilla luciferase reporter using Lipofectamine 3000. After 48 h, the luciferase activity was detected by the dual-luciferase reporter system (Promega) according to manufacturer's instructions.

### 2.12 DNA pull-down assay

BT474-TR and SKBR3-TR cells were trypsinized and washed twice with ice-cold PBS, the nuclear protein was isolated using NE-PER Nuclear and Cytoplasmic Extraction reagents (Thermo Fisher Scientific). Then, the human eIF3j promoter oligonucleotides containing wild-type or mutant ARE were synthesized and labeled with biotin, followed by annealing to form double-stranded oligonucleotides. The above nuclear extracts were incubated with biotinylated DNA probes at 4 °C overnight with gentle rotation, followed by incubation with streptavidin magnetic beads (Invitrogen) for another 1 h. Then, the beads were extensively washed and resuspended in Laemmli buffer, followed by Western blot assay.

### 2.13 Chromatin immunoprecipitation (ChIP)

ChIP assay was performed by using the commercialized SimpleChIP® Plus Sonication Chromatin IP Kit (Cell Signaling Technology) following manufacturer’s protocols. Briefly, BT474-TR and SKBR3-TR cells were cross-linked using 1% formaldehyde, and glycine was added to quench formaldehyde. Cells were then sonicated to obtain chromatin with an average size of 200∼1000 bp, followed by centrifugation at 20,000×*g* for 15min. The supernatant was collected and incubated with anti-NRF2 antibody and protein A/G magnetic beads (Thermo Fisher Scientific) at 4 °C overnight with gentle rotation. The next day, the beads were washed and the precipitated DNA fragments were eluted and analyzed by PCR assay. The primer sequences are listed in Table S2.

### 2.14 Statistical analysis

Data were mean ± standard deviation (SD) of at least three independent experiments carried out in triplicate. Comparisons between the two groups were tested by unpaired *t*-test. Differences among three or more groups were tested by 1-way ANOVA or 2-way ANOVA, followed by Tukey's post hoc test. The survival curve was plotted by Kaplan-Meier curve and tested by log-rank test. The diagnostic efficiency of plasma *circ-β-TrCP* was tested by receiver operating characteristic (ROC) curve and calculated by area under curve (AUC) value. The production of charts and statistics was completed by using GraphPad Prism 8. *P* < 0.05 was considered statistically significant. All *P* values were two-sided unless otherwise specified.

## 3 Results

### 3.1 *circ-β-TrCP* is upregulated in trastuzumab-resistant HER2-positive breast cancer

**Fig. 1:**
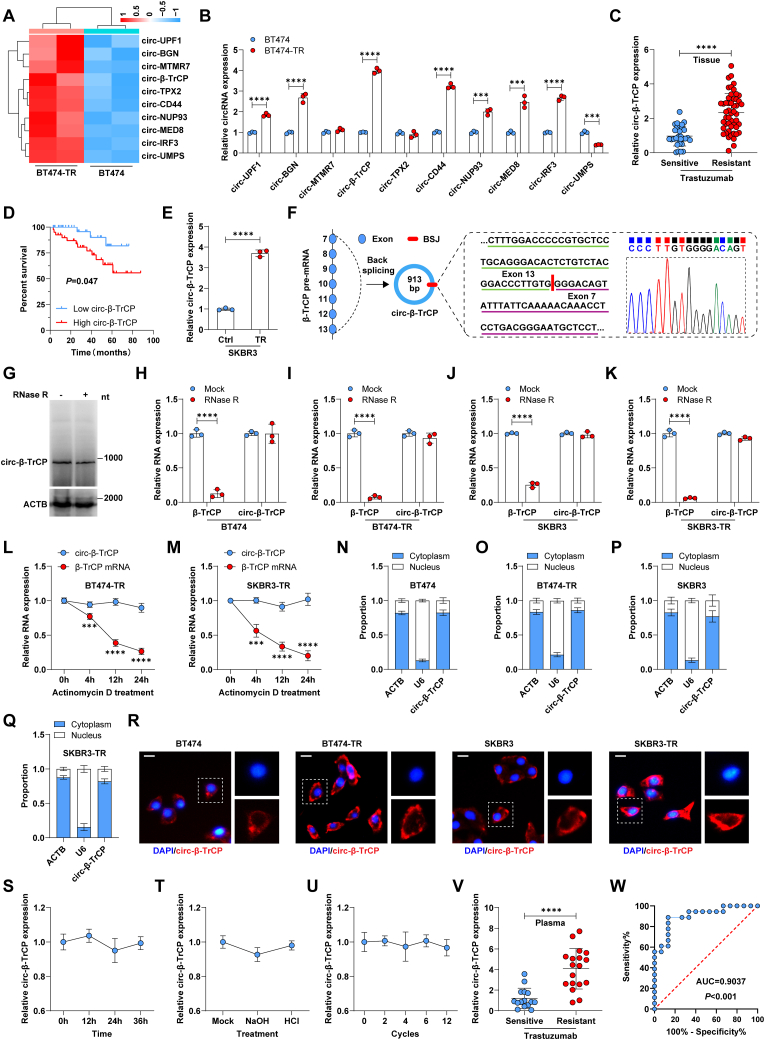
*circ-β-TrCP* is highly expressed in trastuzumab-resistant HER2-positive breast cancer. A. CircRNA sequencing showing the top 10 upregulated circRNAs in BT474-TR cells compared to BT474 cells. B. qRT-PCR analysis of these 10 circRNA levels in BT474-TR and BT474 cells. C. qRT-PCR analysis of *circ-β-TrCP* expression in trastuzumab-sensitive (n = 30) and -resistant (n = 50) HER2-positive breast cancer tissues. D. The survival curve of HER2-positive breast cancer patients with low and high *circ-β-TrCP* based on median *circ-β-TrCP* expression. E. qRT-PCR analysis of *circ-β-TrCP* expression in SKBR3-TR and SKBR3 cells. F. The diagram showing the source of *circ-β-TrCP* while being verified by Sanger sequencing. G. Northern blot testing the full-length of *circ-β-TrCP*. H–K. Cells were treated with or without 5 U/μg RNase R, followed by qRT-PCR analysis of *circ-β-TrCP* and *β-TrCP* mRNA expression. L, M. Cells were treated with 100 μM Actinomycin D for the indicated time, followed by qRT-PCR analysis of *circ-β-TrCP* and β-TrCP mRNA expression. N-Q. qRT-PCR analysis of the location of *circ-β-TrCP*, *ACTB* and *U6* were used as the cytoplasmic and nuclear control fragments, respectively. R. FISH assay testing the location of *circ-β-TrCP*, DAPI was used to stain nucleus. Scale bar = 25 μm. S–U. qRT-PCR analysis of *circ-β-TrCP* expression in plasma after being left at room temperature for different times, acid and alkali treatment and repeated freeze-thawing. V. qRT-PCR analysis of *circ-β-TrCP* expression in plasma from trastuzumab-sensitive (n = 15) and -resistant (n = 18) HER2-positive breast cancer patients. W. ROC curve testing the diagnostic efficiency of plasma *circ-β-TrCP*, analyzed by AUC value. Two-tailed ***P < 0.001, ****P < 0.0001. Unpaired *t*-test was used for B, C, E, H–K, V. 1-way ANOVA followed by Tukey's post hoc test was used for L, M, S–U. Log-rank test was used for D. TR = trastuzumab resistance, ctrl = control, BSJ = back splicing junction.

In our previous study [25], we screened for differential expression of circular RNAs between BT474-TR and BT474 cells. The top 10 upregulated circular RNAs (Fig. 1A) in BT474-TR cells were validated using qRT-PCR assay, the results showed that *circ-β-TrCP* had the largest up-regulation ratio (Fig. 1B), thus we chose it for follow-up study. Consistently, *circ-β-TrCP* was overexpressed in human trastuzumab-resistant breast cancer tissues (Fig. 1C). Patients expressing high *circ-β-TrCP* had shorter overall survival time than those expressing low *circ-β-TrCP* (Fig. 1D). And high *circ-β-TrCP* was also observed in SKBR3-TR cells compared to the parental SKBR3 cells (Fig. 1E). Sequence alignment results showed that *circ-β-TrCP* is derived from the back-splicing of *β-TrCP* pre-mRNA exon 7 and 13, the mature full-length is 913 bp, which was further verified by Sanger sequencing (Fig. 1F) and northern blot (Fig. 1G). After treatment with 5 U/μg RNase R, *β-TrCP* mRNA expression was dramatically reduced, while *circ-β-TrCP* expression was almost unchanged in both trastuzumab-sensitive and -resistant cells (Fig. 1H–K). The half-life of *circ-β-TrCP* exceeded 24 h, while that of *β-TrCP* mRNA was less than 8 h (Fig. 1L and M), suggesting that *circ-β-TrCP* is highly stable. Next, we explored the subcellular localization of *circ-β-TrCP*, the results of qRT-PCR and FISH showed that *circ-β-TrCP* was mainly located in the cytoplasm of both trastuzumab-sensitive and -resistant cells (Fig. 1N–R), indicating that the localization of *circ-β-TrCP* was not altered during trastuzumab resistance. Moreover, *circ-β-TrCP* was also detected in the plasma, and its expression was unaffected by acid-base imbalance, room temperature placement and repeated freeze-thaw (Fig. 1S–U). And plasma *circ-β-TrCP* expression was approximately 4-fold higher in trastuzumab-resistant patients than in sensitive patients (Fig. 1V), with an AUC value of 0.9037 (95% confidence interval (CI): 0.7998–1.000) (Fig. 1W), implying that plasma *circ-β-TrCP* may be an excellent indicator for evaluating patients' response to trastuzumab. Taken together, these data demonstrate that *circ-β-TrCP* is a cytoplasmic circular RNA that may be essential for trastuzumab resistance.

### 3.2 *circ-β-TrCP* promotes trastuzumab resistance

**Fig. 2:**
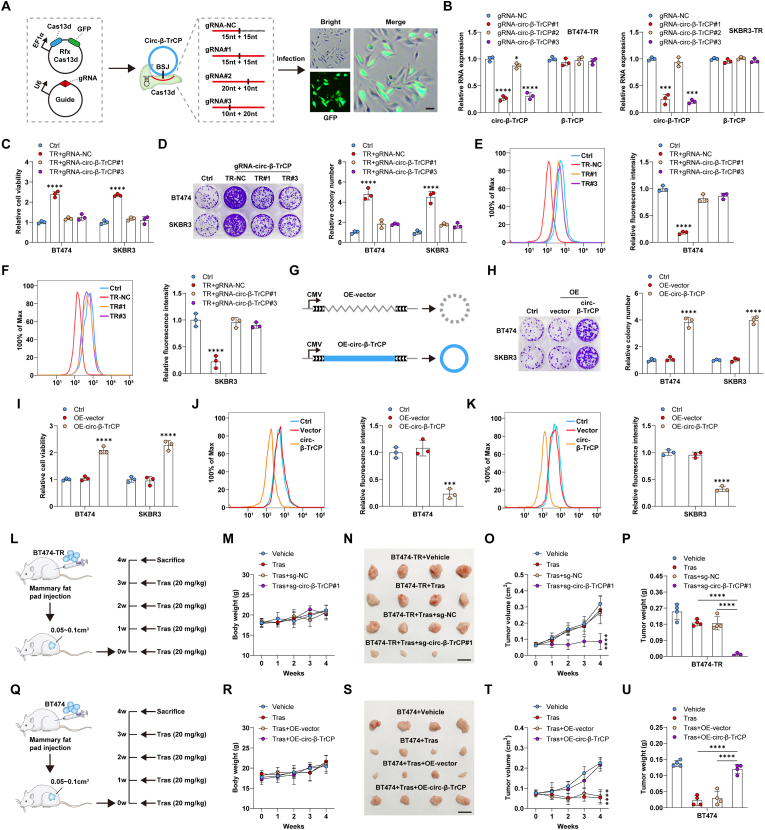
*circ-β-TrCP* drives trastuzumab resistance. A. The diagram showing the use of CRISPR/Cas13d system to silence *circ-β-TrCP*. Scale bar = 25 μm. B. qRT-PCR verifying the silencing efficiency of these designed sgRNAs on *circ-β-TrCP* in BT474-TR and SKBR3-TR cells. C. CCK-8 testing cell viability after *circ-β-TrCP* knockdown following 15 μg/mL trastuzumab treatment. D. Colony formation assay testing cell cloning ability after *circ-β-TrCP* knockdown following 15 μg/mL trastuzumab treatment. E, F. Flow cytometry testing ROS content in *circ-β-TrCP*-silenced BT474-TR and SKBR3-TR cells treated with 15 μg/mL trastuzumab. G. The diagram showing the vector used for *circ-β-TrCP* overexpression. H–K. Cloning ability, cell viability and ROS levels were detected in BT474 and SKBR3 cells transfected with *circ-β-TrCP* overexpressing vector in the presence of 15 μg/mL trastuzumab. L, Q. The diagram showing the establishment of orthotopic transplantation tumor model via mammary fat pad injection of BT474-TR or BT474 cells, followed by intraperitoneal administration of 20 mg/kg trastuzumab once a week for a total of 4 times. M, R. The weight change of mice in the indicated four groups. N–P, S–U. The tumor image, volume and weight in the indicated four groups. Two-tailed *P < 0.05, ***P < 0.001, ****P < 0.0001. 1-way ANOVA followed by Tukey's post hoc test was used for B–F, H–K, P, U. 2-way ANOVA followed by Tukey's post hoc test was used for M, O, R, T. BSJ = back splicing junction, NC = negative control, TR = trastuzumab resistance, ctrl = control, denotes BT474 and SKBR3 cells that have not received any treatment, OE = overexpression, Tras = trastuzumab.

Next, we used CRISPR/Cas13d system to knock down endogenous *circ-β-TrCP* in trastuzumab-resistant cells, and established stable cell lines (Fig. 2A). Among the 3 designed sgRNAs targeting *circ-β-TrCP* junction site, gRNA-*circ-β-TrCP*#1 and #3, but not #2, were able to substantially knock down *circ-β-TrCP*, and did not affect linear *β-TrCP* mRNA expression (Fig. 2B). The results of CCK-8 showed that cell viability was significantly reduced after silencing of *circ-β-TrCP* following trastuzumab treatment (Fig. 2C). Less colony formation was observed in *circ-β-TrCP*-silenced BT474-TR and SKBR3-TR cells compared to control cells treated with trastuzumab (Fig. 2D). And *circ-β-TrCP*-silenced BT474-TR and SKBR3-TR cells exhibited increased ROS levels following trastuzumab treatment, as illustrated by H2DCFDA and CellROX staining (Figs. S1A and B, Fig. 2E and F). Further, we overexpressed *circ-β-TrCP* in BT474 and SKBR3 cells (Fig. 2G), colony formation (Fig. 2H) and cell viability (Fig. 2I) were notably enhanced, while ROS levels (Fig. 2J, K, Fig. S1C) were decreased in the presence of trastuzumab.

The orthotopic transplantation tumor model was established to assess the *in vivo* effect of *circ-β-TrCP* on trastuzumab resistance (Fig. 2L). BT474-TR cells were orthotopically injected into the abdominal mammary fat pad of NOD/SCID mice, followed by a weekly dosing of trastuzumab (Fig. 2L). All mice survived at the end of the treatment, and no mouse exhibited severe loss of body weight (>15%) or evidence of infections or wounds (Fig. 2M). The results showed that BT474-TR-derived tumors were highly resistant to trastuzumab (Fig. 2N–P), however, tumor was evidently smaller after knockdown of *circ-β-TrCP* (Fig. 2N–P). In contrast, BT474-derived tumor size was reduced following trastuzumab administration (Fig. 2Q–U), while overexpression of *circ-β-TrCP* significantly attenuated the therapeutic effect of trastuzumab (Fig. 2Q–U). In all, these *in vitro* and *in vivo* results suggest that *circ-β-TrCP* is a driver of trastuzumab resistance.

### 3.3 *circ-β-TrCP* contributes to trastuzumab resistance via encoding β-TrCP-343aa

**Fig. 3:**
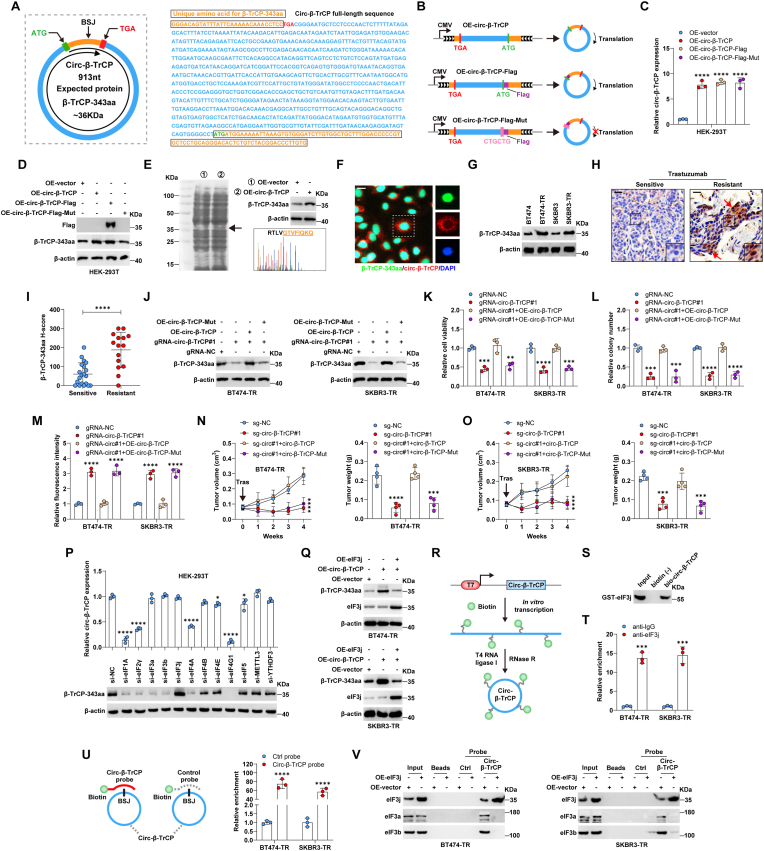
*circ-β-TrCP* encodes β-TrCP-343aa, which is repressed by eIF3j. A. The diagram showing the full-length sequence of *circ-β-TrCP* and its predicted encoding peptide. B. The diagram showing the construction of OE-*circ-β-TrCP*, OE-*circ-β-TrCP*-Flag and OE-*circ-β-TrCP*-Flag with mutation of start codon. C. qRT-PCR analysis of *circ-β-TrCP* expression in HEK-293T cells transfected with the indicated vectors. D. Western blot testing the protein levels of Flag and β-TrCP-343aa in HEK-293T cells transfected with the indicated vectors. E. Coomassie brilliant blue staining of *circ-β-TrCP*-overexpressed cell lysates, followed by mass spectrometry identification. The black arrow indicates the location where the gel was cut for mass spectrometry. F. The fluorescent signals showing the subcellular localization of *circ-β-TrCP* and β-TrCP-343aa, nucleus was stained by DAPI. Scale bar = 25 μm. G. Western blot testing the protein expression of β-TrCP-343aa in the indicated cells. H. The representative images of IHC staining of β-TrCP-343aa in trastuzumab-resistant and -sensitive HER2-positive breast cancer tissues. The red arrows denote the representative staining. Scale bar = 50 μm. I. Semi-quantitative analysis of β-TrCP-343aa IHC staining using H-score method. J. Western blot testing β-TrCP-343aa protein expression in *circ-β-TrCP*-silenced BT474-TR and SKBR3-TR cells transfected with the indicated vectors. K-M. Detection of cell viability, colony formation and ROS content in *circ-β-TrCP*-silenced BT474-TR and SKBR3-TR cells transfected with the indicated vectors following 15 μg/mL trastuzumab treatment. N, O. Tumor volume and weight in the indicated four groups. P. HEK-293T cells were transfected with siRNA targeting the indicated genes, followed by qRT-PCR and Western blot detecting *circ-β-TrCP* and β-TrCP-343aa expression, respectively. Q. Western blot detecting β-TrCP-343aa and eIF3j protein levels in *circ-β-TrCP*-overexpressed BT474-TR and SKBR3-TR cells transfected with eIF3j expressing vector. R. The diagram showing the *in vitro* synthesis of full-length of *circ-β-TrCP*. S. RNA pull-down coupled with Western blot testing the *in vitro* binding of *circ-β-TrCP* to eIF3j. T. RIP assay using anti-eIF3j antibody, followed by qRT-PCR analysis of *circ-β-TrCP* enrichment. U. The diagram showing the design of *circ-β-TrCP* probe targeting the junction site of *circ-β-TrCP*, followed by qRT-PCR analysis verifying the enrichment efficiency of *circ-β-TrCP* probe. V. RNA pull-down assay in eIF3j-overexpressed BT474-TR and SKBR3-TR cells using *circ-β-TrCP* probe, followed by Western blot analysis of protein expression of eIF3j, eIF3a and eIF3b. Two-tailed *P < 0.05, **P < 0.01, ***P < 0.001, ****P < 0.0001. 1-way ANOVA followed by Tukey's post hoc test was used for C, K-M, N (right panel), O (right panel), P. Unpaired *t*-test was used for I, T, U. 2-way ANOVA followed by Tukey's post hoc test was used for N (left panel), O (left panel). BSJ = back splicing junction, OE = overexpression, Mut = mutation, NC = negative control, TR = trastuzumab resistance, ctrl = control, Tras = trastuzumab.

Through analyzing TransCirc database [29], we found that *circ-β-TrCP* potentially encodes a 343-amino acid protein across the junction site with a predicted molecular weight size of 36 KDa, named as β-TrCP-343aa (Fig. 3A). Due to the lack of a stop codon in the first-round read, β-TrCP-343aa has 28 and 10 unique amino acids at the 5′amino and 3′carboxy terminals, respectively (Fig. 3A). To test whether *circ-β-TrCP* is translatable, we inserted the Flag sequence after the start codon ATG, or mutated “ATGATG” to “CTGCTG” (Fig. 3B), which did not affect the expression of *circ-β-TrCP* (Fig. 3C). As shown in Fig. 3D, the Flag band was detected near 35 KDa, whereas mutation of the start codon did not produce Flag protein. The existence of β-TrCP-343aa translated by *circ-β-TrCP* was further verified by mass spectrometry (Fig. 3E). Next, we generated a rabbit polyclonal antibody against the 3′carboxy terminal (VGTVFIQKQTS) of β-TrCP-343aa. This antibody successfully recognized endogenous β-TrCP-343aa (Fig. 3D and E), and the protein signal was significantly enhanced in cells transfected with *circ-β-TrCP*-overexpressed vector, but not vector with start codon mutation (Fig. 3D). The fluorescence results showed that *circ-β-TrCP* and β-TrCP-343aa were predominantly located in the cytoplasm and nucleus, respectively (Fig. 3F). Moreover, β-TrCP-343aa was overexpressed in trastuzumab-resistant cells and tissues in comparison to trastuzumab-sensitive controls (Fig. 3G–I). Silencing of *circ-β-TrCP* dramatically reduced endogenous β-TrCP-343aa levels in BT474-TR and SKBR3-TR cells, while these effects were blocked by overexpression of *circ-β-TrCP*, but not by *circ-β-TrCP* with start codon mutation (Fig. 3J). Functionally, overexpression of wild-type *circ-β-TrCP*, not the mutated one without translation ability, abolished the reduced cell growth *in vitro* (Fig. 3K) and *in vivo* (Fig. 3N and O), colony formation (Fig. 3L) and increased ROS content (Fig. 3M) caused by *circ-β-TrCP* knockdown following trastuzumab treatment. These data suggest that *circ-β-TrCP* coding product, rather than *circ-β-TrCP* itself, endows cells with trastuzumab resistance.

Studies have shown that some translation initiation factors and N6-methyladenosine (m^6^A) regulators are responsible for circRNA translation [30]. We then used RNAi screening to identify the factors governing the translation process of *circ-β-TrCP*. The results of qRT-PCR and Western blot showed that knockdown of *eIF3* subunits evidently affected β-TrCP-343aa production, while had little effects on the expression of *circ-β-TrCP* (Fig. 3P), hinting that *eIF3* is involved in *circ-β-TrCP* translation, but not *circ-β-TrCP* biogenesis. Specifically, silencing of *eIF3a* and *eIF3b* decreased, while silencing of *eIF3j* increased β-TrCP-343aa levels (Fig. 3P), which is consistent with the findings showing that *eIF3j* repressed circRNA translation through inhibiting translation initiation via blocking the binding of eIF3a and eIF3b to circRNA [31]. And the increased β-TrCP-343aa caused by *circ-β-TrCP* overexpression was effectively blocked by *eIF3j* overexpression in both BT474-TR and SKBR3-TR cells (Fig. 3Q). The *in vitro* binding assay was conducted using synthetic full-length *circ-β-TrCP* and purified eIF3j protein, the results showed that *circ-β-TrCP* directly binds to eIF3j (Fig. 3R and S). Furthermore, the endogenous interaction between *circ-β-TrCP* and eIF3j protein was verified by RIP and RNA pull-down assays (Fig. 3T and U). As shown in Fig. 3V, eIF3a,eIF3b and eIF3j proteins were all pulled by *circ-β-TrCP* probes in both BT474-TR and SKBR3-TR cells, whereas less or almost no eIF3a and eIF3b proteins were observed to be interacted with *circ-β-TrCP* in the presence of eIF3j overexpression. These results indicate that *circ-β-TrCP* translation is negatively regulated by eIF3j.

### 3.4 β-TrCP-343aa blocks SCF^β-TRCP^-mediated degradation of NRF2

**Fig. 4:**
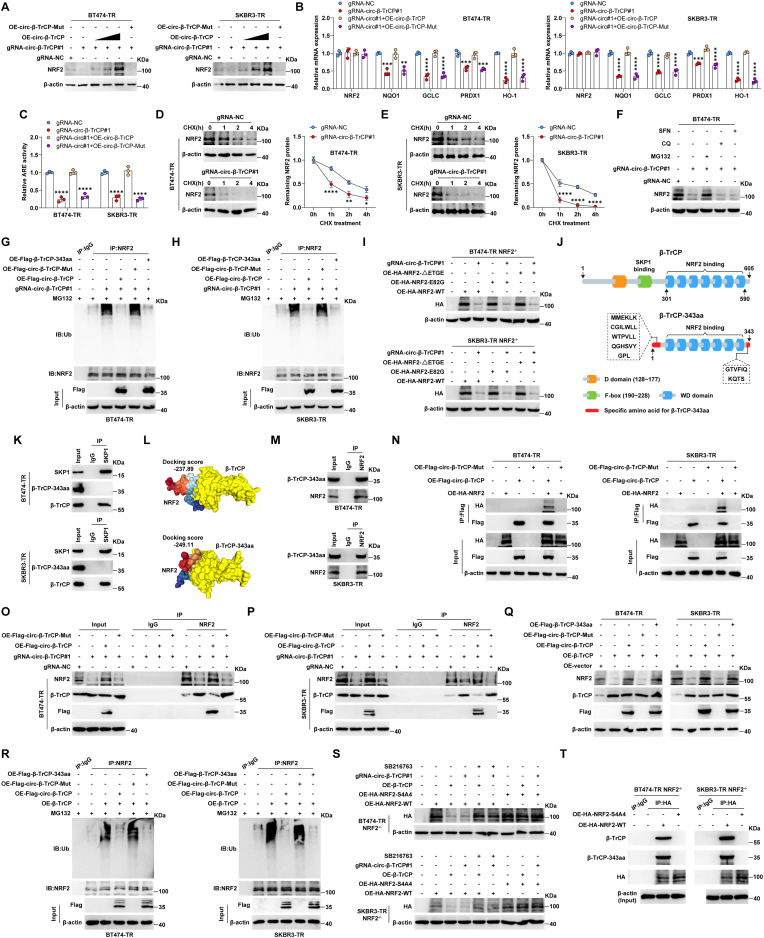
β-TrCP-343aa blocks SCF^β-TRCP^-mediated degradation of NRF2. A. Western blot testing the protein expression of NRF2 in *circ-β-TrCP*-silenced BT474-TR and SKBR3-TR cells transfected with *circ-β-TrCP* expressing vector or *circ-β-TrCP* expressing vector with start codon mutation. B. qRT-PCR analysis of the expression of NRF2 targets in *circ-β-TrCP*-silenced BT474-TR and SKBR3-TR cells transfected with the indicated vectors. C. ARE-luciferase reporter assay detecting *NRF2* activity in *circ-β-TrCP*-silenced BT474-TR and SKBR3-TR cells transfected with the indicated vectors. D, E. Western blot testing NRF2 protein expression in *circ-β-TrCP*-silenced BT474-TR and SKBR3-TR cells after treatment with 100 μg/mL cycloheximide for the indicated time. F. Western blot testing NRF2 protein expression in *circ-β-TrCP*-silenced cells treated with 20 μM sulforaphane, 10 μM chloroquine, 20 μM MG132. G, H. IP assay using anti-NRF2 antibody, followed by Western blot analysis of ubiquitin expression in *circ-β-TrCP*-silenced BT474-TR and SKBR3-TR cells transfected with the indicated vectors. I. Western blot testing HA protein expression in NRF2^−/−^ cells transfected with the indicated vectors. J. The cartoon showing the specific protein domains of β-TrCP and β-TrCP-343aa. K. IP assay using anti-SKP1 antibody in BT474-TR and SKBR3-TR cells, followed by Western blot detecting SKP1, β-TrCP and β-TrCP-343aa protein levels. L. The protein docking models of β-TrCP-NRF2 and β-TrCP-343aa-NRF2. M. IP assay using anti-NRF2 antibody in BT474-TR and SKBR3-TR cells, followed by Western blot detecting NRF2 and β-TrCP-343aa protein levels. N. IP assay using anti-Flag affinity gels in BT474-TR and SKBR3-TR cells transfected with the indicated plasmids, followed by Western blot analysis of Flag and HA levels. O, P. IP assay using anti-NRF2 antibody in *circ-β-TrCP*-silenced cells transfected with the indicated plasmids, followed by Western blot analysis of NRF2, β-TrCP and Flag levels. Q. Cells were transfected with the indicated vectors, followed by Western blot analysis of NRF2, β-TrCP and Flag levels. R. IP assay using anti-NRF2 antibody, followed by Western blot analysis of ubiquitin expression in β-TrCP-overexpressed BT474-TR and SKBR3-TR cells transfected with the indicated vectors. S. NRF2^−/−^ cells were transfected with the indicated vectors or treated with 10 μM SB216763, followed by Western blot analysis of HA expression. T. IP assay using anti-HA affinity gels in NRF2^−/−^ cells transfected with HA-NRF2-WT or HA-NRF2-S4A4 (S344A/S347A/S351A/S356A), followed by Western blot analysis of β-TrCP, β-TrCP-343aa and HA levels. Two-tailed *P < 0.05, **P < 0.01, ***P < 0.001, ****P < 0.0001. 1-way ANOVA followed by Tukey's post hoc test was used for B, C. 2-way ANOVA followed by Tukey's post hoc test was used for D, E. OE = overexpression, Mut = mutation, NC = negative control, TR = trastuzumab resistance, CHX = cycloheximide, SFN = sulforaphane, CQ = chloroquine, WT = wild-type.

Given that β-TrCP-343aa contains the WD structural domain of β-TrCP, and β-TrCP degrades nuclear NRF2, a key factor in trastuzumab resistance, we thus inferred that β-TrCP-343aa might function via affecting the β-TrCP/NRF2 axis. As expected, knockdown of *circ-β-TrCP* in BT474-TR and SKBR3-TR cells significantly reduced NRF2 protein levels, which was rescued by restoration of *circ-β-TrCP* expression in a dose-dependent manner (Fig. 4A), but not by overexpression of *circ-β-TrCP* with start codon mutation (Fig. 4A). And the mRNA levels of NRF2 targets, *NQO1*, *GCLC*, *PRDX1* and *HO-1*, as well as *NRF2* transcriptional activity, were reduced in *circ-β-TrCP*-silenced cells compared to control cells, and these effects were also counteracted by *circ-β-TrCP* overexpression (Fig. 4B and C). Of note, the abundance of *NRF2* mRNA was unchanged (Fig. 4B), suggesting that β-TrCP-343aa modulates NRF2 at the post-transcriptional level. After blocking protein synthesis with 100 μg/mL cycloheximide (CHX), the degradation rate of NRF2 protein was significantly accelerated after *circ-β-TrCP* knockdown (Fig. 4D and E). The decreased NRF2 protein caused by *circ-β-TrCP* silencing was blocked by MG132 (a proteasome inhibitor), but not by chloroquine (CQ, an autophagy inhibitor) (Fig. 4F), suggesting that the ubiquitin-proteasome system is involved in β-TrCP-343aa-mediated regulation of NRF2 protein turnover. Consistently, the ubiquitination levels of NRF2 were markedly increased in *circ-β-TrCP*-silenced cells, and these effects were blocked by overexpression of *circ-β-TrCP* or linear β-TrCP-343aa, but not by *circ-β-TrCP* without translation ability (Fig. 4G and H). Moreover, knockdown of *circ-β-TrCP* was able to reduce NRF2 protein levels in the presence of sulforaphane (SFN), a NRF2 activator modifying the critical cystine residues of KEAP1 to repress NRF2 ubiquitination (Fig. 4F). And both NRF2 ETGE deletion and E82G variants, which disrupt the binding of KEAP1 to NRF2, failed to rescue *circ-β-TrCP* knockdown-mediated NRF2 downregulation in NRF2^−/−^ cells (Fig. 4I). These data suggest that β-TrCP-343aa stabilizes NRF2 protein independent of KEAP1.

By comparing β-TrCP and β-TrCP-343aa protein sequences, it was found that they both have WD domain that binds to NRF2, but β-TrCP-343aa lacks F-box domain that recruits SKP1-Cul1-Rbx1 complex to degrade NRF2 (Fig. 4J). Consistently, the IP results showed that β-TrCP, but not β-TrCP-343aa, was immunoprecipitated by SKP1 (Fig. 4K). Thus, we speculated that β-TrCP-343aa might compete with β-TrCP to bind to NRF2, resulting in NRF2 not being degraded. The protein docking model shows that β-TrCP-343aa has a higher NRF2 binding capacity than β-TrCP (Fig. 4L). And the endogenous interaction between β-TrCP-343aa and NRF2 was verified by IP assay in both BT474-TR and SKBR3-TR cells (Fig. 4M). Moreover, exogenous HA-NRF2 was also immunoprecipitated by Flag-β-TrCP-343aa translated by *circ-β-TrCP* (Fig. 4N). As expected, *circ-β-TrCP* knockdown led to more NRF2 binding to β-TrCP, accompanied by a decrease in NRF2 protein expression, and these phenomena were abrogated by *circ-β-TrCP* overexpression, but not by *circ-β-TrCP* harboring start codon mutation (Fig. 4O and P). Besides, NRF2 protein levels were decreased, while the ubiquitination levels were increased in β-TrCP-overexpressing BT474-TR and SKBR3-TR cells, the above effects were blocked by introduction of *circ-β-TrCP* or linear β-TrCP-343aa, but not by the mutated *circ-β-TrCP* (Fig. 4Q and R). It is known that β-TrCP binding to the Neh6 domain of NRF2 requires the degron phosphorylated by GSK3, we then wondered whether this was also the case for the binding of β-TrCP-343aa to NRF2. As shown in Fig. 4S, overexpression of β-TrCP or knockdown of *circ-β-TrCP* significantly reduced NRF2 levels in NRF2^−/−^cells reexpressing HA-NRF2 rather than HA-NRF2-S4A4 (Alanine substitution of four serines within the Neh6 phosphodegron of NRF2), and these phenomena disappeared after treatment with the GSK3 inhibitor SB216763. Furthermore, the IP results showed that NRF2-S4A4 failed to bind to β-TrCP and β-TrCP-343aa (Fig. 4T).

Collectively, these findings demonstrate that *circ-β-TrCP*-encoded β-TrCP-343aa directly binds to and stabilizes NRF2 protein through inhibiting the β-TrCP-NRF2 interaction, which is independent of the KEAP1 pathway but requires GSK3 activity.

### 3.5 NRF2 increases the translation efficiency of *circ-β-TrCP* under normal and oxidative stress conditions via transcriptional inhibition of eIF3j

**Fig. 5:**
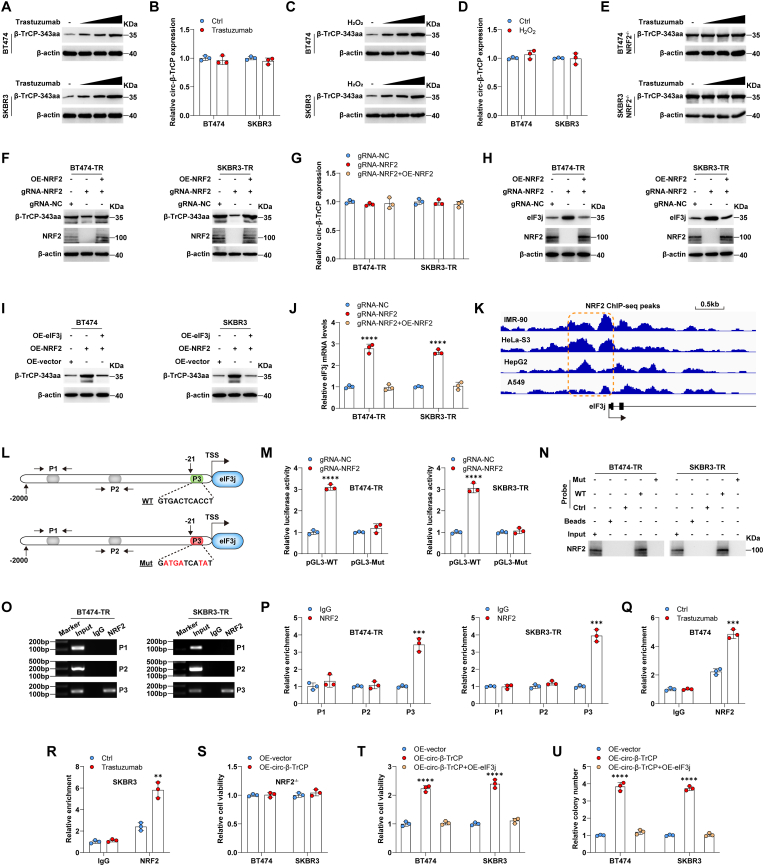
NRF2 increases the translation efficiency of *circ-β-TrCP* via transcriptional inhibition of eIF3j. A-D. BT474 and SKBR3 cells were treated with trastuzumab and H_2_O_2_, followed by Western blot and qRT-PCR analysis of β-TrCP-343aa and *circ-β-TrCP* expression, respectively. E. Western blot testing β-TrCP-343aa protein expression in NRF2^−/−^ BT474 and SKBR3 cells treated with trastuzumab. F–H. Western blot and qRT-PCR testing β-TrCP-343aa/eIF3j protein and *circ-β-TrCP* expression, respectively, in NRF2^−/−^ BT474-TR and SKBR3-TR cells transfected with NRF2 expressing plasmid. I. Western blot testing β-TrCP-343aa protein expression in NRF2-overexpressed BT474 and SKBR3 cells transfected with eIF3j expressing plasmid. J. qRT-PCR analysis of eIF3j mRNA expression in NRF2^−/−^ BT474-TR and SKBR3-TR cells transfected with NRF2 expressing plasmid. K. The NRF2 ChIP-seq peaks tested in IMR-90, HeLa–S3, HepG2 and A549 cells from ENCODE database. L. The diagram showing the location of ARE and primers designing for ChIP assay in eIF3J promoter. M. Luciferase reporter assay detecting eIF3j promoter activity in NRF2^−/−^ BT474-TR and SKBR3-TR cells transfected with wild-type or mutant eIF3j promoter reporters. N. DNA pull-down assay using wild-type or mutant eIF3j promoter probe in BT474-TR and SKBR3-TR cell nucleus extracts, followed by Western blot testing NRF2 protein levels. O, P. ChIP assay using anti-NRF2 antibody, followed by PCR analysis using the above designed primers. Q, R. ChIP assay using anti-NRF2 antibody in BT474 and SKBR3 cells treated with 15 μg/mL trastuzumab, followed by PCR analysis of ARE enrichment. S. CCK-8 testing cell viability in NRF2^−/−^ BT474 and SKBR3 cells transfected with *circ-β-TrCP* expressing plasmid. T, U. Cell viability and colony formation were tested in *circ-β-TrCP*-overexpressed BT474 and SKBR3 cells transfected with eIF3j expressing plasmid. Two-tailed **P < 0.01, ***P < 0.001, ****P < 0.0001. Unpaired *t*-test was used for B, D, P, S. 1-way ANOVA followed by Tukey's post hoc test was used for G, J, T, U. 2-way ANOVA followed by Tukey's post hoc test was used for M, Q, R. Ctrl = control, OE = overexpression, NC = negative control, TR = trastuzumab resistance, Mut = mutation, WT = wild-type.

Notably, trastuzumab or H_2_O_2_ treatment increased β-TrCP-343aa levels in a dose-dependent manner, however, the expression of *circ-β-TrCP* was unaltered (Fig. 5A–D), hinting that *circ-β-TrCP* translation, but not *circ-β-TrCP* biogenesis, is regulated in response to oxidative stress. Trastuzumab increased ROS levels in BT474 and SKBR3 cells (Fig. S2A), but not in BT474-TR and SKBR3-TR cells (Fig. S2B), and β-TrCP-343aa expression remained unchanged following trastuzumab or H_2_O_2_ treatment in NRF2^−/−^cells (Fig. 5E), suggesting that NRF2 is critical for β-TrCP-343aa production under oxidative stress conditions. Knockout of NRF2 reduced β-TrCP-343aa expression (Fig. 5F), but not *circ-β-TrCP* expression (Fig. 5G), and this effect was rescued by reintroduction of NRF2 in BT474-TR and SKBR3-TR cells (Fig. 5F), indicating that NRF2 also controls β-TrCP-343aa expression under basal conditions. Further, eIF3j protein was upregulated in NRF2^−/−^cells compared to wild-type NRF2 cells, while decreased by restoration of NRF2 expression (Fig. 5H). And the increased β-TrCP-343aa levels caused by NRF2 overexpression was antagonized by simultaneous overexpression of eIF3j (Fig. 5I), indicating that eIF3j participates in NRF2-mediated regulation of β-TrCP-343aa. Moreover, the mRNA levels of *eIF3j*, like its protein levels, were also modulated by NRF2 (Fig. 5J), hinting the transcriptional regulation of NRF2 on eIF3j.

Through analyzing the NRF2 ChIP-seq data in ENCODE datasets [32], we found that numerous NRF2 peaks are located in the promoter region of *eIF3j* (Fig. 5K). Further, one ARE is found at −21 ∼ −11 region in *eIF3j* promoter using JASPAR online tool [33](Fig. 5L). We then mutated the ARE and conducted luciferase reporter assay, the results showed that NRF2 knockout significantly increased wild-type *eIF3j* promoter activity, while did not affect the mutant one (Fig. 5M). Likewise, NRF2 protein in nucleus extracts of BT474-TR and SKBR3-TR cells was enriched by wild-type *eIF3j* promoter probe, but not by the mutated probe (Fig. 5N). Next, we designed three pairs of primers targeting the indicated regions in *eIF3j* promoter (P1 and P2 were used as negative controls, P3 contained the ARE) (Fig. 5L), followed by ChIP assay to test the binding of NRF2 to *eIF3j* promoter *in vivo*. As shown in Fig. 5O, the fragments pulled down by NRF2 were only amplified by P3, but not by P1 and P2. And the qRT-PCR results showed that NRF2 enrichment on ARE motif in *eIF3j* promoter was nearly 4-fold higher than IgG (Fig. 5P). To further confirm that this ARE is critical for NRF2 binding to *eIF3j* promoter, we employed CRISPR/Cas9 genome editing and generated endogenous eIF3j ARE-mutated SKBR3 cell lines by mutating six nucleotides from GTGACTCACCT to GATGATCATAT (Fig. S3A). As shown in Fig. S3B, mutation of *eIF3j* ARE abolished the binding of NRF2 to *eIF3j* promoter, even when NRF2 was overexpressed. And NRF2 was not able to reduce *eIF3j* expression in *eIF3j* ARE-mutated cells (Fig. S3C). Moreover, trastuzumab treatment remarkably increased the occupation of NRF2 on *eIF3j* promoter (Fig. 5Q and R). Functionally, overexpression of *circ-β-TrCP* did not affect cell viability following trastuzumab treatment in NRF2^−/−^cells (Fig. 5S), and the enhanced cell viability and colony formation caused by *circ-β-TrCP* overexpression in the presence of trastuzumab were significantly abolished by eIF3j introduction (Fig. 5T and U), indicating that *circ-β-TrCP* promotes trastuzumab resistance via the NRF2/*eIF3j* axis.

Altogether, these data suggest that NRF2 accelerates the translation process of *circ-β-TrCP* via transcriptionally repressing of *eIF3j*, thus forming a positive regulatory loop between β-TrCP-343aa and NRF2, expediting trastuzumab resistance.

## 4 Discussion

**Fig. 6:**
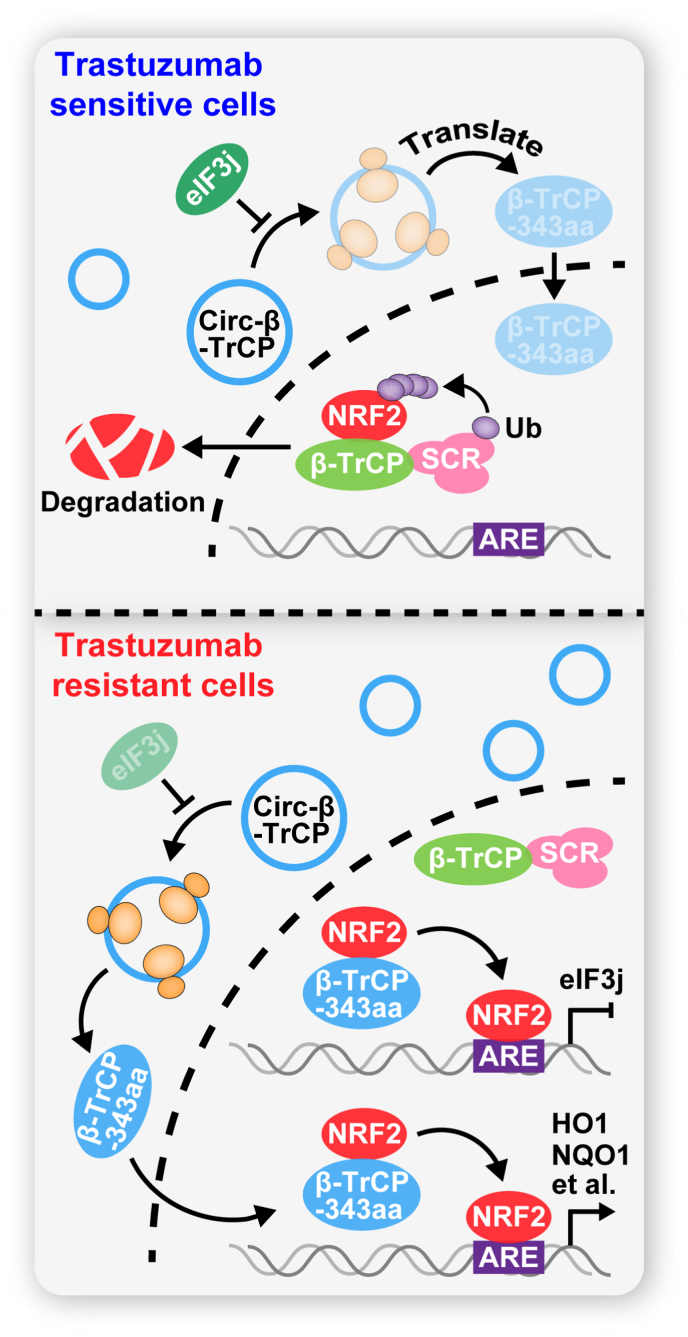
The proposed model showing that *circ-β-TrCP* encodes β-TrCP-343aa, then β-TrCP-343aa competes with SKP1-Cul1-Rbx1 (SCR)-β-TrCP complex to bind to NRF2 in the nucleus, leading to NRF2 stabilization and transcriptionally elevation of antioxidant genes, thereby promoting trastuzumab resistance. However, in trastuzumab-sensitive cells, *circ-β-TrCP* translating into β-TrCP-343aa is repressed by *eIF3j*, NRF2 is bound by β-TrCP, followed by NRF2 ubiquitination degradation, and the transcriptional repressive effect of NRF2 on *eIF3j* is blocked.

The emergence of drug resistance has greatly limited the clinical use of trastuzumab. In this study, we found that *circ-β-TrCP* plays an essential role in trastuzumab resistance. Genetic intervention of *circ-β-TrCP* expression significantly altered the sensitivity of breast cancer cells to trastuzumab *in vitro* and *in vivo*. Further studies revealed that *circ-β-TrCP* contributes to trastuzumab resistance in a NRF2-dependent manner. *circ-β-TrCP* encodes a novel β-TrCP protein isoform, β-TrCP-343aa, that competes with β-TrCP to bind to NRF2 in the nucleus, thereby blocking the ubiquitination degradation of NRF2 mediated by SCF^β-TrCP^ E3 complex (Fig. 6). Moreover, *circ-β-TrCP* translating into β-TrCP-343aa is inhibited by *eIF3j* under both normal and oxidative stress conditions, and NRF2 is able to directly bind to *eIF3j* promoter and inhibit its transcription, thus a positive loop is formed between β-TrCP-343aa and NRF2, ultimately accelerating trastuzumab resistance (Fig. 6). Therefore, our work provides new evidence for the importance of the coding function of circular RNA in cell biology, as well as new insights into the mechanisms of trastuzumab resistance in HER2-positive breast cancer patients.

Biomarkers are indicators of disease changes or treatment effects through the detection of proteins and genes contained in human blood, urine and other body fluids or tissues [34]. It can be used not only to select therapeutic drugs for tumors, but also to determine the presence or absence of tumors or metastases. Due to the cell- or tissue-specific expression pattern, high conservatism and stability, circular RNA is considered to be an excellent biomarker to monitor tumor occurrence, development and progression [35,36]. For example, *circ-NEIL3*, a *TGFβ*-repressive circular RNA, was identified as a potential biomarker for colorectal carcinoma liver metastasis [37]. Hepatocellular carcinoma patients with high *circ-SORE* had poor recurrence-free survival, and its tissues content was proposed as a good predictor of sorafenib efficacy [38]. Also, circular RNA is demonstrated to be enriched and stable in human peripheral blood [39,40]. *F-circEA* [41], *circ-CCAC1* [42], *circ-N4BP2L2* [43] and *circ-RNF220* [44] were abundantly expressed in human peripheral blood, which could be used as the biomarkers for diagnosis of *EML4-ALK*-positive non-small cell lung cancer, cholangiocarcinoma, epithelial ovarian cancer and pediatric acute myeloid leukemia, respectively. Herein, we found that HER2-positive breast cancer patients harboring high *circ-β-TrCP* displayed worse overall survival than those harboring low *circ-β-TrCP*, indicating that *circ-β-TrCP* may be a prognostic biomarker for HER2-positive breast cancer patients. More importantly, *circ-β-TrCP* was also significantly upregulated in plasma from trastuzumab-resistant patients compared to trastuzumab-sensitive patients, with an AUC value of 0.9037, suggesting that plasma *circ-β-TrCP* has great potential to be used as a non-invasive indicator of trastuzumab response.

NRF2 is a well-known antioxidant that confers cellular resistance to anti-cancer drugs, including trastuzumab [45]. Elucidating the regulatory network of NRF2 will shed new light on drug resistance. Under normal physiological conditions, the protein abundance of NRF2 is low, due to rapid degradation by KEAP1 [46,47]. However, in response to oxidative stress, KEAP1 undergoes conformational changes and loses NRF2 binding capacity, causing NRF2 accumulation [48]. Several factors have been reported to play key roles in disease progression and drug resistance by regulating NRF2 protein level in a KEAP1-dependent or -independent manner, such as ATDC [49], TRIM22 [50] and REDD1 [51]. These findings mainly focused on studying the regulation of NRF2 protein turnover in the cytoplasm, however, little is known about its stability regulation in the nucleus, that is where NRF2 exerts the antioxidant effects. It is well documented that nuclear NRF2 protein is degraded by SCF^β-TrCP^ E3 complex [52], but how this process is regulated and whether it involves substantial nuclear accumulation of NRF2 under oxidative stress is not yet clear. In this study, we found a novel β-TrCP isoform, β-TrCP-343aa, translated by *circ-β-TrCP*, which has the WD domain that binds to NRF2, but has no F-box domain of β-TrCP that binds to SKP1-Cul1-Rbx1 complex and ubiquitinates NRF2. Thus, it was speculated that β-TrCP-343aa inhibits β-TrCP-mediated NRF2 degradation via competitively binding to NRF2. Protein docking model, IP and Western blot assays confirmed this hypothesis that β-TrCP-343aa is a previously unrecognized stabilizer of nuclear NRF2. And under oxidative stress, β-TrCP-343aa is substantially elevated dependent of NRF2, thus a positive feedback loop is formed between them, which can be used to explain why NRF2 rapidly and substantially accumulates in the nucleus in response to oxidative stress. Of note, the phosphodegron created by GSK-3 on NRF2 Neh6 domain is necessary for the binding of β-TrCP to NRF2 [53], a condition that also applies to the interaction between β-TrCP-343aa and NRF2, as illustrated by the loss of β-TrCP-343aa regulation of NRF2 protein stability when the activity of GSK3 was inhibited. Hence, GSK3 activity is a prerequisite for the protective effect of β-TrCP-343aa on nuclear NRF2 protein.

NRF2 is a transcription factor that forms heterodimers with sMaf proteins in the nucleus to recognize the ARE, followed by recruitment of chromatin remodeling complexes and coactivators to transcriptionally active a battery of genes involved in anti-stress responses [54]. In addition to regulation of redox homeostasis, NRF2 is also involved in various cellular processes, including metabolic reprogramming, proteostasis, immunity, inflammation, and more [55]. Thus, the target gene network of NRF2 is far more complex than we currently know and requires in-depth dissection, which may lead to new insights into disease onset and progression. Here, we identified a novel target of NRF2. NRF2 was capable to directly bind to the ARE located at −21 ∼ −11 region in eIF3j promoter, thereby inhibiting eIF3j transcription. Mutation of this ARE abolished the transcriptional repressive effect of NRF2 on eIF3j, and more NRF2 occupied the promoter of eIF3j during oxidative stress, suggesting that eIF3j is a bona fide target gene of NRF2 under both basal and oxidative stress conditions. It is worth noting that the NRF2-sMaf heterodimers typically activates gene transcription, thus, this model is not applicable to the regulation of eIF3j by NRF2. A study showed that NRF2-replication protein A1 element (NRE) adjacent to the 3′-end of the ARE recognized by NRF2-RPA1 heterodimers is critical for the transcriptional repressive effect of NRF2 [56], however, no NRE was found flanking ARE in eIF3j promoter. The precise mechanism responsible for NRF2-mediated eIF3j repression remains to be defined and may involve inhibition of RNA Pol II recruitment [57]or cooperation with chromatin-modifying enzymes such as EZH2 [58].

There are several limitations in this work, with the major drawback being that the *in vitro* and *in vivo* studies were performed using cell lines and do not truly reflect clinical tumor heterogeneity and the interactions between tumor cells and microenvironment, the use of patient-derived xenograft and organoids models will be helpful. In addition, the sample size we studied is relatively small, which may result in potential bias, further large-scale multi-center studies are needed to determine the diagnostic and prognostic efficacy of *circ-β-TrCP*. Meanwhile, whether *circ-β-TrCP* exists in other human fluids such as sweat, urine, saliva, as well as whether it is transported by some vesicles like exosomes, are worthy of in-depth investigation.

Collectively, our data uncover a novel mechanism of trastuzumab resistance driven by circular RNA-encoded peptides, meanwhile provide a proof-of-concept demonstration for a potential strategy to resensitize trastuzumab-resistant HER2-positive breast cancer patients to trastuzumab.

## Author contributions

STW, YFW and QL performed all functional experiments. STW and KXZ performed data acquisition and interpretation. XML performed animal experiments. XHF provided theoretical and technical guidance. KXZ wrote this manuscript, conceived and supervised the entire project. All authors read, revised and approved the final manuscript.

## Declaration of competing interest

The authors declare that they have no known competing financial interests or personal relationships that could have appeared to influence the work reported in this paper.

## Data Availability

Data will be made available on request.
