# Supplementary material for: The novel β-TrCP protein isoform hidden in circular RNA confers trastuzumab resistance in HER2-positive breast cancer
Source: Redox Biol. 2023 Sep 28;67:102896. doi: 10.1016/j.redox.2023.102896 (PMC10551893; doi:10.1016/j.redox.2023.102896)
Supplement: Multimedia component 1 [file mmc1.pdf]

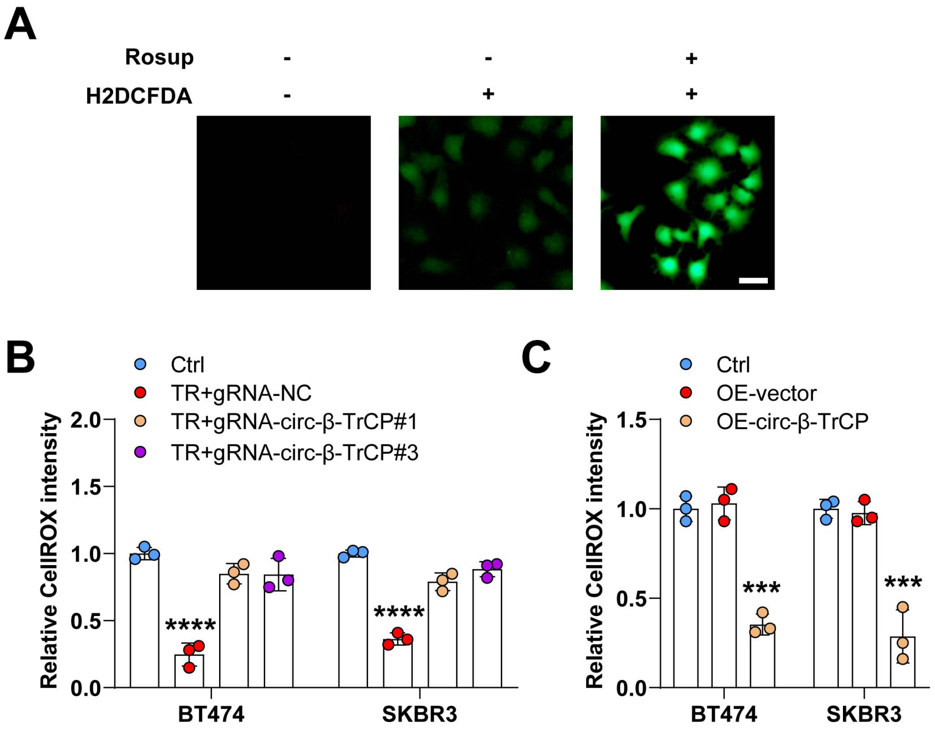

Figure S1. A. Verification of the efficiency of H2DCFDA for detecting ROS levels. Rosup (50μg/mL), a ROS inducer, was used as the positive control. Scale bar=25μm. B. CellROX staining testing ROS levels in *circ-β-TrCP*-silenced BT474-TR and SKBR3-TR cells treated with 15 μg/mL trastuzumab. C. CellROX staining testing ROS levels in BT474 and SKBR3 cells transfected with *circ-β-TrCP* overexpressing vector in the presence of 15 μg/mL trastuzumab. Two-tailed ****P*<0.001, *****P*<0.0001. Ctrl=control, denotes BT474 and SKBR3 cells that have not received any treatment. NC=negative control, TR=trastuzumab resistance.

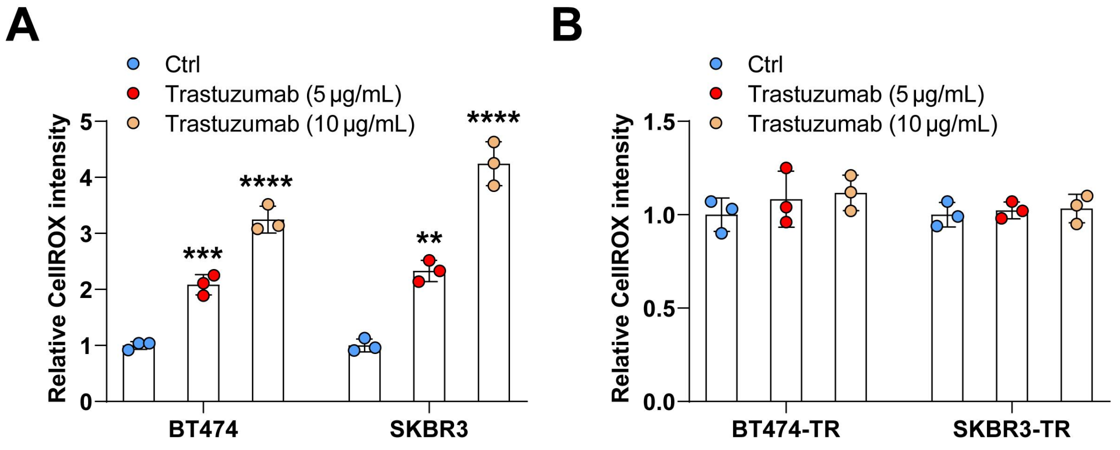

Figure S2. CellROX staining testing ROS levels in BT474, SKBR3, BT474-TR and SKBR3-TR cells treated with the indicated concentrations of trastuzumab. Two-tailed ***P*<0.01, ****P*<0.001,*****P*<0.0001.

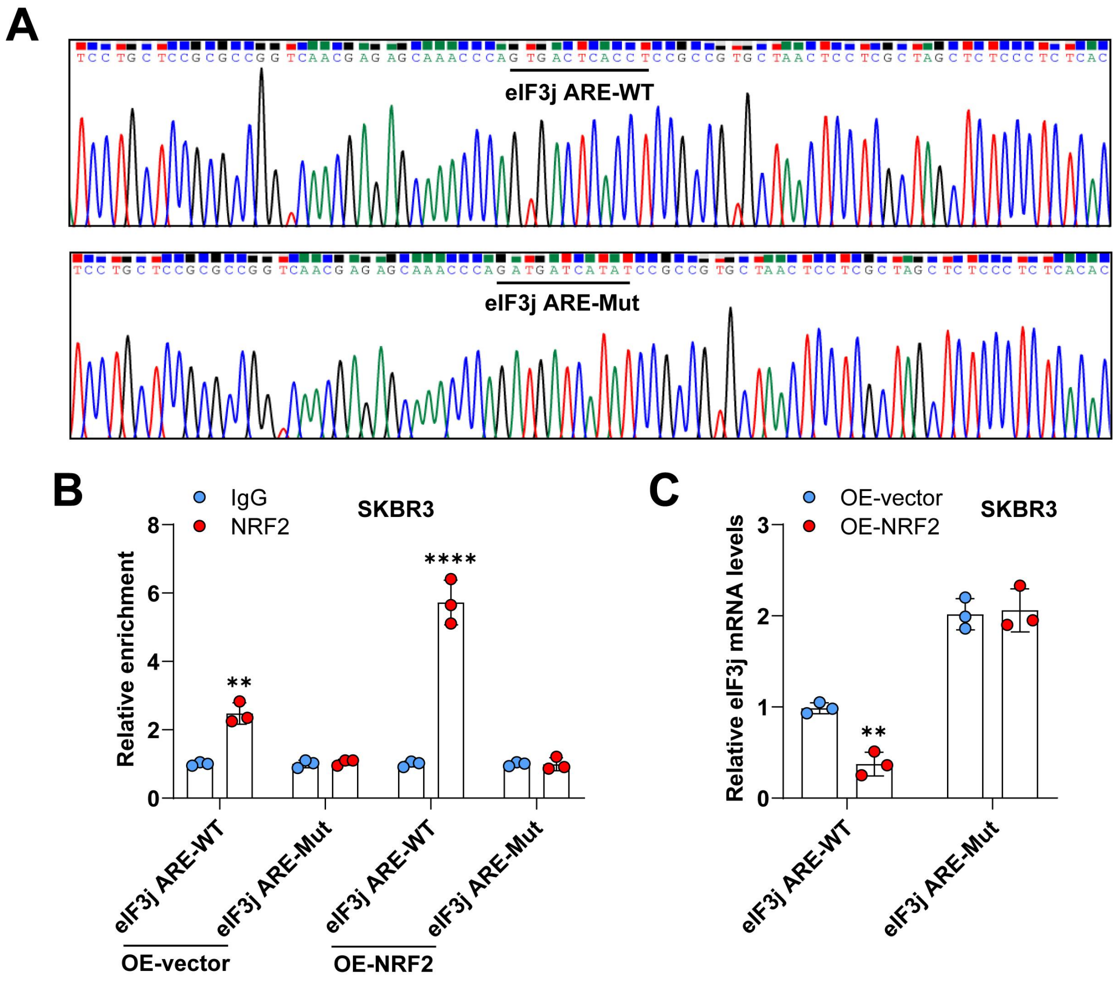

Figure S3. A. Sanger sequencing verifying successful construction of endogenous eIF3j ARE-mutated SKBR3 cells. B. ChIP assay using anti-NRF2 antibody in wild-type or mutant eIF3j-ARE SKBR3 cells transfected with NRF2-overexpressing plasmid. C. qRT-PCR analysis of eIF3j mRNA expression in wild-type or mutant eIF3j-ARE SKBR3 cells transfected with NRF2-overexpressing plasmid. Two-tailed $^{**}P<0.01$, $^{****}P<0.0001$.
